# Retropharyngeal Hematoma under Rivaroxaban: A Rare Entity to Know for Its Risk of Airway Obstruction

**DOI:** 10.5334/jbsr.2263

**Published:** 2021-03-16

**Authors:** Charlotte Vierendeels, Xavier Peeters, Pierre Bosschaert

**Affiliations:** 1 Clinique Saint-Pierre Ottignies, BE

**Keywords:** Retropharyngeal, Hematoma, Dysphagia, Rivaroxaban, Trauma

## Abstract

**Teaching Point:** Retropharyngeal hematoma appearing under rivaroxaban is uncommon but should be suspected in cases of dysphagia, dysphonia or breathing difficulties.

## Case

A 74-year-old man was admitted to the emergency department for progressive dysphagia and mild dysphonia in the context of minor bicycle trauma that had occurred four days earlier. He had no significant swelling nor any cervical spine pain and no limitation of neck movement. He had no signs of airways being compromised. Previous medical history included anticoagulation with rivaroxaban for atrial fibrillation and aortic valve replacement four months before the bicycle trauma. Examination of the oropharynx was unremarkable. He was afebrile, with normal blood pressure.

**Figure 1:**
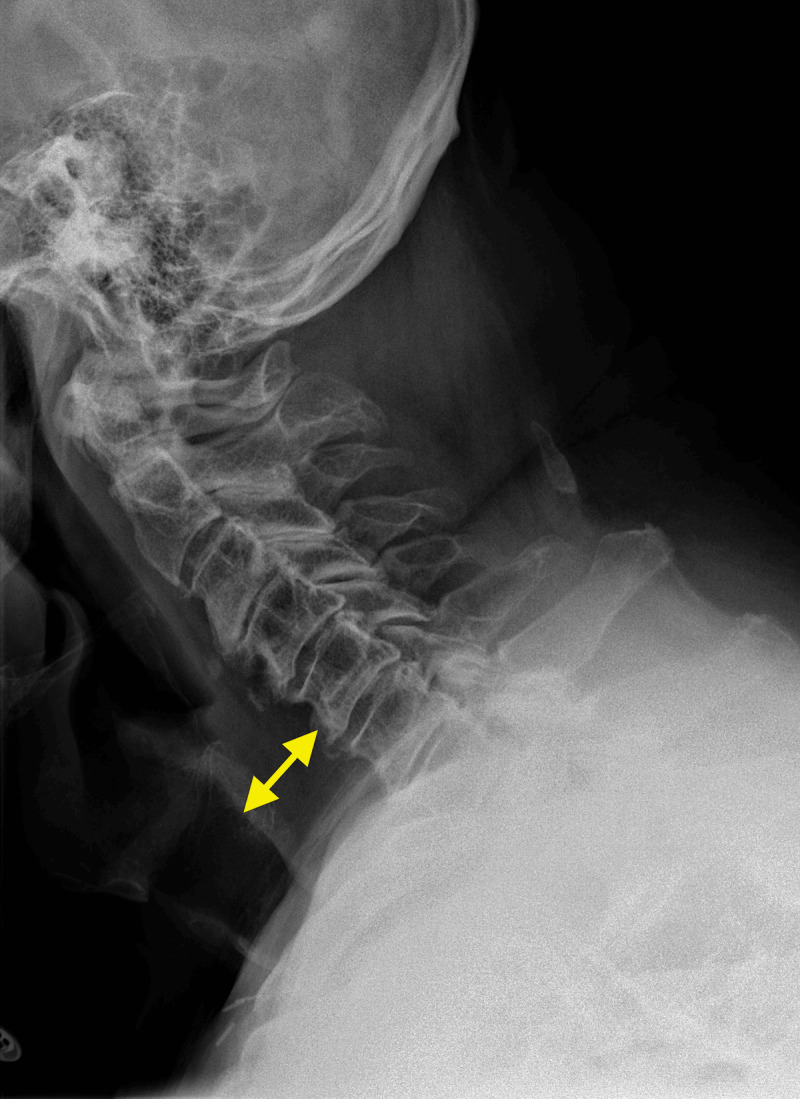
Lateral cervical X-ray showing soft tissue thickness of 30 mm in front of C5 (double arrow).

**Figure 2:**
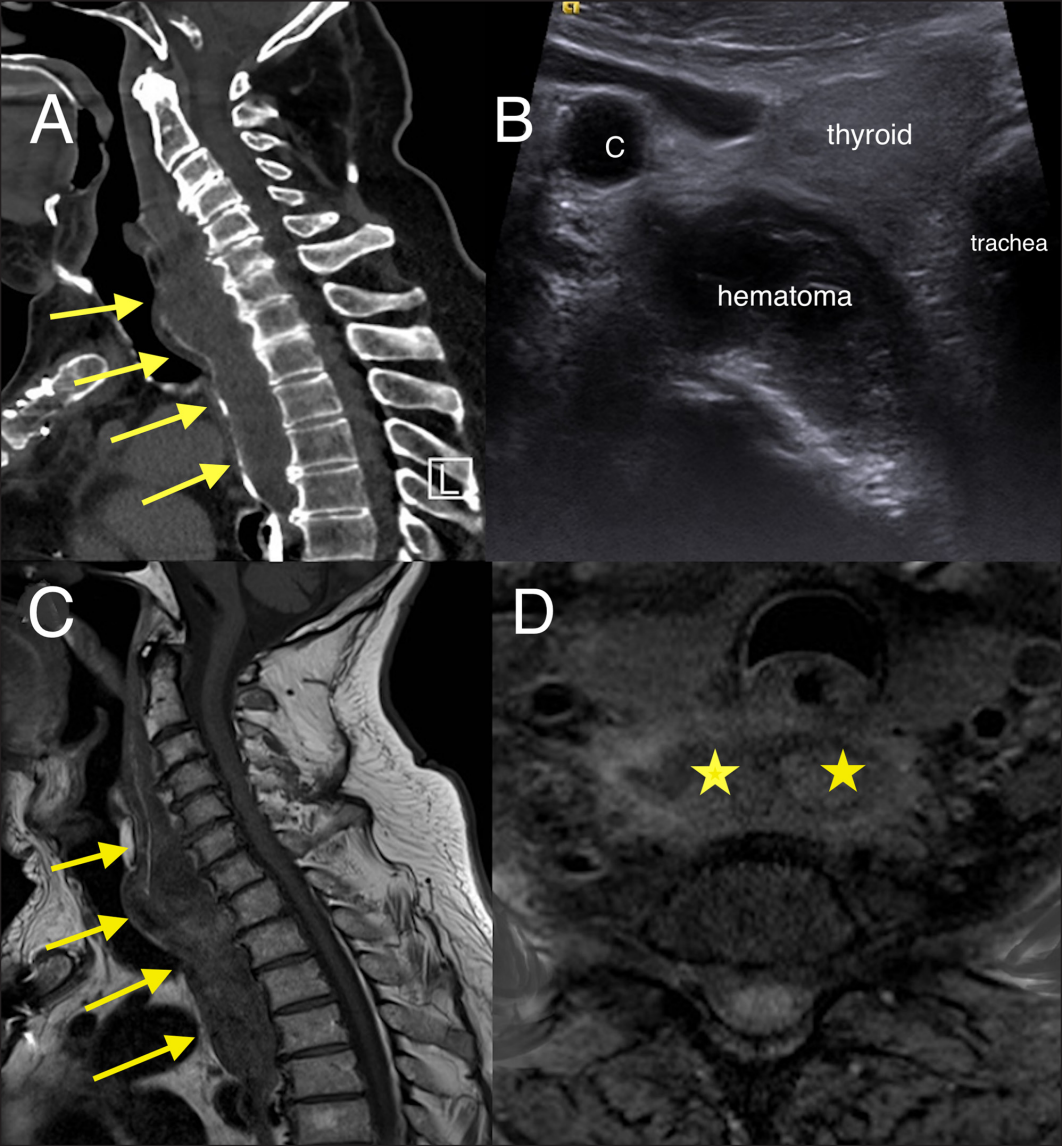
**(A)** Sagittal CT image with oral gastrografin administration showing a pre-vertebral hematoma displacing the pharynx (arrows). **(B)** Axial US image showing the slightly echoic collection pushing forward trachea and thyroid. **(C)** Sagittal T1-weighted image and **(D)** Axial T1-weighted image with fat saturation showing the retropharyngeal hematoma on day 7 with methemoglobin hyperintensity (arrows and asterisks).

Lateral cervical X-ray showed increased soft tissue thickness of 30 mm in front of C5 (***Figure 1***, double arrow). Contrast-enhanced computed tomography (CECT) with oral gastrografin administration revealed hematoma extending from the retropharyngeal to the superior mediastinal space without active bleeding or vertebral fracture (***Figure 2A***, arrows). Axial US image showed a slightly echoic and heterogeneous liquid collection pushing forward trachea and thyroid (***Figure 2B***).

The hematoma was not drained due to its good tolerance. The patient was monitored closely. On magnetic resonance imaging (MRI) performed seven days later, T1-weighted sagittal (***Figure 2C***, arrows) and axial sequence with fat saturation (***Figure 2D***, stars) confirmed methemoglobin hyperintensity. A control MRI carried out three months later showed the disappearance of the hematoma.

## Comment

Retropharyngeal hematoma is a rare entity that can cause airway obstruction rapidly with a potentially fatal outcome. The diagnosis is often delayed because of the absence of objective signs and diagnostic laboratory data.

Many circumstances can lead to its development, such as a trauma, intra-thyroid bleeding, hemophilia, and more rarely in the context of rivoxaraban medication [1].

The onset is usually acute but can also be insidious, and patients may present symptoms only several days after hematoma has developed. Patients with symptoms such as dysphagia, dysphonia, or dyspnea should receive immediate attention.

The diagnosis is suggested by thickening of the pre-vertebral space on lateral cervical X-ray. CECT is the examination of first choice in order to confirm and measure the retropharyngeal hematoma. Patients may also show active bleeding. Any associated vertebral fracture may also be ruled out. Ultrasound (US) can be done at the patient’s bedside. MRI offers the advantage of anatomical precision and is recommended for follow-up.

Non-compressive hematoma can be treated conservatively by cervical spine immobilization and close clinical monitoring. For a retropharyngeal hematoma producing significant airway obstruction, a tracheostomy may be necessary. Surgical evacuation is generally reserved for hematomas that develop quickly, obstruct mechanical ventilation, or do not resolve themselves traditionally.
